# Therapeutic Approaches and Potential Mechanisms of Small Extracellular Vesicles in Treating Vascular Dementia

**DOI:** 10.3390/cells14060409

**Published:** 2025-03-11

**Authors:** Yujie Yang, Chunchu Deng, Fatima Aldali, Yunjie Huang, Hongmei Luo, Yizhou Liu, Danxia Huang, Xiaojian Cao, Qiuzhi Zhou, Jia Xu, Yajie Li, Hong Chen

**Affiliations:** 1 Department of Rehabilitation, Tongji Hospital, Tongji Medical College, Huazhong University of Science and Technology, Wuhan 430030, China; m202276342@hust.edu.cn (Y.Y.); deng_c@tjh.tjmu.edu.cn (C.D.); fatima.aldali12@gmail.com (F.A.); m202376557@hust.edu.cn (Y.H.); luohongmei@hust.edu.cn (H.L.); m202176104@hust.edu.cn (Y.L.); d202282137@hust.edu.cn (D.H.); zephyrus@hust.edu.cn (X.C.); 2023tj0332@hust.edu.cn (Q.Z.); jiaxuz0604@hust.edu.cn (J.X.); 2018tj5463@hust.edu.cn (Y.L.); 2 Stem Cell Research Center, Tongji Hospital, Tongji Medical College, Huazhong University of Science and Technology, Wuhan 430030, China; 3 Hubei Key Laboratory of Neural Injury and Functional Reconstruction, Huazhong University of Science and Technology, Wuhan 430030, China

**Keywords:** small extracellular vesicles, vascular dementia, pathogenesis, therapeutic strategies, treatment

## Abstract

Small extracellular vesicles (sEVs), including exosomes as a subtype, with a diameter typically less than 200 nm and originating from the endosomal system, are capable of transporting a diverse array of bioactive molecules, including proteins, nucleic acids, and lipids, thereby facilitating intercellular communication and modulating cellular functions. Vascular dementia (VaD) represents a form of cognitive impairment attributed to cerebrovascular disease, characterized by a complex and multifaceted pathophysiological mechanism. Currently, the therapeutic approach to VaD predominantly emphasizes symptom management, as no specific pharmacological treatment exists to cure the condition. Recent investigations have illuminated the significant role of sEVs in the pathogenesis of vascular dementia. This review seeks to provide a comprehensive analysis of the characteristics and functions of sEVs, with a particular focus on their involvement in vascular dementia and its underlying mechanisms. The objective is to advance the understanding of the interplays between sEVs and vascular dementia, thereby offering novel insights for future research and therapeutic strategies.

## 1. Introduction

Vascular dementia, a condition resulting from cerebrovascular disorders, is marked by progressive cognitive decline, neurodegeneration, and memory impairments [1]. It is prevalent among the elderly population, with its incidence rising linearly with advancing age [2]. Presently, vascular dementia is recognized as the second most prevalent form of dementia, following Alzheimer’s disease, accounting for at least 20% of cases [3]. Epidemiological research has delineated multiple risk factors associated with vascular dementia (VaD), such as hypertension, hyperlipidemia, diabetes mellitus, smoking, and cardiovascular diseases, thereby highlighting the critical need for preventive measures and early intervention strategies [4,5].

Contemporary therapeutic strategies for vascular dementia (VaD) predominantly focus on addressing vascular risk factors and enhancing cerebral blood flow [6,7]. Pharmacological treatments, including acetylcholinesterase inhibitors and memantine, have demonstrated limited effectiveness in the management of VaD [8]. Additionally, non-pharmacological interventions, such as cognitive rehabilitation and lifestyle modifications, are integral components of the treatment framework [3,9,10]. Nevertheless, these therapeutic approaches present significant limitations. Pharmacological agents aimed at alleviating cognitive symptoms in vascular dementia (VaD) frequently provide only modest benefits and are often accompanied by adverse effects. In contrast, non-pharmacological interventions, while potentially beneficial, may necessitate substantial resources and may not be feasible for all patients. Moreover, there are currently no specific disease-modifying treatments available for VaD, nor are there effective strategies to decelerate or reverse the damage associated with this condition [11]. Consequently, comprehending the pathogenesis of vascular dementia and identifying effective strategies for its prevention and treatment are of paramount importance [12].

The pathophysiological mechanisms underlying vascular dementia are intricate, encompassing the multifaceted interplay between vascular and neurodegenerative factors. Chronic cerebral hypoperfusion, small vessel disease, and microvascular injury collectively contribute to neuronal damage and white matter degeneration, thereby impairing cognitive function [13,14]. Furthermore, an increased permeability of the blood–brain barrier and the dysregulation of cerebral blood flow result in the accumulation of neurotoxic waste products, exacerbating cerebral damage [15,16]. Additionally, neuroinflammation and oxidative stress are two intertwined processes in the pathogenesis of vascular dementia [17]. Microglia and astrocyte activation in neuroinflammation triggers the release of inflammatory mediators [18]. These mediators not only directly damage neurons but also disrupt the blood–brain barrier, facilitating the entry of harmful substances and further aggravating the disease [19,20]. Meanwhile, oxidative stress, resulting from an imbalance in free radical production and scavenging, elevates intracellular free radical levels [21]. This leads to cellular damage, apoptosis, and the impairment of neuronal survival, thereby promoting neurodegeneration and worsening cognitive function [22,23]. Notably, oxidative stress can activate the neuroinflammatory response, and neuroinflammation in turn can exacerbate oxidative stress by generating more reactive oxygen species (ROS) [24]. The two processes interact with each other, forming a vicious cycle that accelerates the progression of vascular dementia [25].

Small extracellular vesicles (sEVs) have emerged as important mediators in the pathophysiology of neurodegenerative diseases, including VaD [26,27]. They can transport bioactive molecules between cells, influencing the cellular environment and disease progression [28,29]. Within the context of VaD, sEVs serve a dual role, where they act as carriers for the regulation of pathological signals and hold potential as therapeutic agents [30,31]. It should be noted that exosomes, a subset of sEVs, have often been misidentified in previous studies. When referring to vesicles with an unclear subcellular origin, it is more accurate to use the term “sEVs”. This review adheres to the updated MISEV rules to ensure the proper use of terminology.

## 2. Overview of Small Extracellular Vesicles

### 2.1. Release and Composition of sEVs

**Figure 1:**
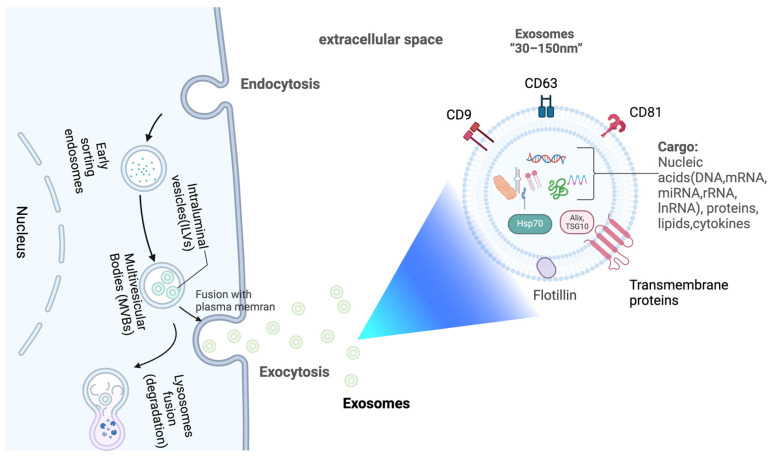
Release and composition of exosomes. Created with BioRender.com.

sEVs are extracellular vesicles with diameters often less than 200 nm [32]. They en-capsulate various genetic materials like mRNA, miRNA, lncRNA, DNA, along with proteins and lipids [33]. Exosomes, a subset of sEVs, are formed through a specific biogenesis process. Early endosome formation marks the beginning of exosome biogenesis [34,35]. Plasma membrane folding leads to the formation of early endosomes, which subsequently undergo a series of intracellular maturation processes to develop into late endosomes. These late endosomes eventually give rise to multivesicular bodies (MVBs) containing intraluminal vesicles (ILVs) [34,36,37,38]. MVBs are capable of fusing with lysosomes, resulting in complete degradation. Conversely, when MVBs fuse with the plasma membrane, they release their intraluminal vesicles (ILVs) via exocytosis thus forming exosomes (Figure 1) [29,39,40]. sEVs are found in a wide range of bodily fluids, including saliva, urine, blood, cerebrospinal fluid, amniotic fluid, and breast milk [41]. The density of sEVs has been reported to vary between 1.13 and 1.19 g/mL [42]. sEVs are characterized by the presence of certain membrane proteins such as CD63, CD9, and CD81 (tetraspanins), along with Alix and TSG101, proteins related to the endosomal sorting complexes required for transport, or ESCRT, involved in the synthesis of multivesicular bodies, and the proteins HSP70 and HSP90, known as heat shock proteins. Examples of these proteins are considered sEV marker proteins due to their prevalent abundance [43,44].

### 2.2. Biological Function of sEVs

As carriers of bioactive molecules, sEVs are important for the communication between cells [34,45]. This communication fosters the exchange of information and signaling molecules, thereby regulating a myriad of physiological and pathological processes [46,47]. As conveyors of immune response, sEVs regulate immune responses by transporting immunomodulatory molecules such as cytokines, chemokines, and microRNAs to immune cells [48,49,50]. They are involved in antigen presentation, immune cell activation, and the establishment of immune tolerance, thereby influencing immune surveillance and inflammatory responses [51,52,53]. sEVs contribute to tissue repair and regeneration through different strategies, such as promoting cell multiplication, migration, and differentiation, overseeing extracellular matrix modification, lessening inflammation, and encouraging angiogenesis [54,55,56,57]. Furthermore, sEVs have gained recognition as a potential source of biomarkers for disease diagnosis, prognosis, and monitoring. These nanovesicles encapsulate a diverse array of biologically active molecules, such as proteins, nucleic acids, lipids, and metabolites, which mirror the physiological and pathological conditions of their originating cells [58,59]. Due to their distinct molecular profiles, sEVs display disease-specific attributes and can be identified and examined in various bodily fluids, offering a non-invasive approach for biomarker discovery [60,61,62].

### 2.3. Advantages of sEVs Compared to Source Cells

sEVs present several advantages over their parental cells in therapeutic and diagnostic applications [63]. In comparison to the original cells, sEVs exhibit enhanced safety profiles as they do not proliferate, thereby mitigating risks such as immune rejection and tumorigenesis that are often associated with cell therapy [64]. Furthermore, sEVs are devoid of cell surface antigens and maintain a natural cell membrane structure, which contributes to their low immunogenicity and diminishes the probability of eliciting immune responses upon administration [34,65]. sEVs encapsulate bioactive molecules within a lipid bilayer membrane, thereby affording protection against enzymatic degradation and environmental factors, which enhances their stability [66,67]. These vesicles can be derived from a variety of cell types, including mesenchymal stem cells [68], dendritic cells [69], and tumor cells [70], thus providing a diverse array of cellular sources for the loading of therapeutic cargo and the achievement of targeting specificity. Furthermore, sEVs are able to be perpetually produced by cells that can divide without limit, thereby ensuring a sufficient supply [71]. They are also readily storable [72], with the ability to be preserved at −80 °C for prolonged durations [66]. sEVs exhibit significant tissue penetration capabilities and can traverse the blood–brain barrier, facilitating the focused transport of medicinal compounds to specific tissues or organs [73].

## 3. The Role of sEVs in the Treatment of Vascular Dementia

### 3.1. Vascular Injury

**Figure 2:**
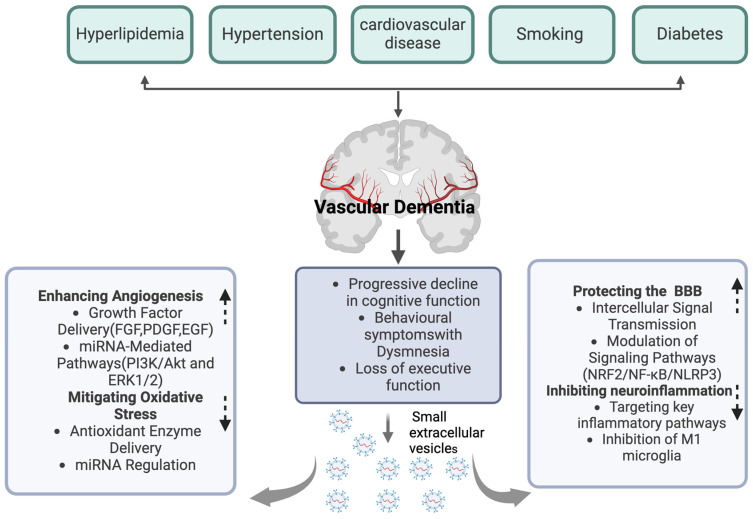
The role of sEVs in the treatment of vascular dementia. Created with BioRender.com.

Vascular injury constitutes a critical pathological mechanism in vascular dementia (VaD), which is characterized by damage to the cerebral blood vessels, thereby diminishing cerebral blood flow and oxygenation, ultimately culminating in cognitive deficits [74,75,76]. sEVs are integral to promoting vascular recovery, thereby contributing positively to the alleviation of vascular dementia (Figure 2) (Table 1). Research indicates that sEVs harbor potent angiogenic paracrine effectors, which facilitate the repair of ischemic tissue-related pathologies by stimulating the production of angiogenic proteins [77]. Notably, mesenchymal stem cell (MSC)-derived sEVs have been demonstrated to enhance angiogenesis through multiple pathways. These sEVs are enriched with growth factors and cytokines, comprising the epidermal growth factor (EGF), fibroblast growth factor (FGF), and platelet-derived growth factor (PDGF), which are essential for facilitating endothelial cell proliferation and migration [77]. Furthermore, sEVs contribute to neovascularization by modulating angiogenesis-related signaling pathways. Specifically, human umbilical cord blood-derived sEVs stimulate angiogenesis by triggering the PI3K/Akt and ERK1/2 signaling pathways via miR-21-3p, thereby expediting the healing of skin wounds [78]. MiR-26a, which is enriched in SHED aggregate-derived sEVs, facilitates angiogenesis in SHED and HUVEC by adjusting the TGF-β/SMAD2/3 signaling pathway [79]. MSC-derived sEVs have been shown to facilitate angiogenesis in human brain microvascular endothelial cells (HBMECs) by upregulating ICAM1 expression and activating the SMAD3 and P38MAPK signaling pathways [80]. Furthermore, the study identified that the miRNA components within sEVs may play an important part in managing angiogenesis. Ratajczak et al. demonstrated that the sEVs extracted from CD133+ cells express mRNAs of several pro-angiogenic factors, such as the kit ligand, insulin-like growth factor-1, vascular endothelial growth factor, basic fibroblast growth factor, and interleukin-8, thereby facilitating angiogenesis both in vitro and in vivo [81]. Similarly, Min Gong and colleagues verified that MSC-derived sEVs, containing pro-angiogenic microRNAs such as miR-30b, miR-30c, miR-424, and let-7f, can boost the expression of pro-angiogenic factors [82]. Furthermore, the study identified that endothelial cells require miR-214 for the secretion of sEVs, which play a role in inhibiting cellular aging and promoting angiogenesis, thereby underscoring the critical role of miRNA in the process of angiogenesis [83].

### 3.2. Blood–Brain Barrier Dysfunction

In vascular dementia, the disruption of the blood–brain barrier (BBB) function leads to disordered metabolite transport across the BBB, thereby jeopardizing brain health and adversely affecting cognitive function [84,85]. sEVs are integral to the amelioration of BBB dysfunction (Figure 2) (Table 2). They can penetrate the BBB through several mechanisms. One primary method is receptor-mediated endocytosis [86]. sEVs express specific ligands on their surface that can bind to receptors on the endothelial cells of the BBB [87]. For instance, some sEVs carry integrin proteins, which can interact with receptors on the BBB endothelial cells, promoting the internalization of sEVs into the cells [88]. Another key mechanism involves the fusion of sEVs with the cell membrane [89]. The lipid bilayer of sEVs can directly fuse with the plasma membrane of BBB endothelial cells, releasing their contents into the cells and thus influencing BBB function [90]. Empirical evidence indicates that sEVs facilitate intercellular signal transmission by transporting specific microRNAs, thereby influencing the structural integrity and functional capacity of the BBB. For instance, research utilizing a mouse model of middle cerebral artery occlusion (MCAO) demonstrated that endothelial cells can receive considerable quantities of microRNAs, like miR-132-3p, via MSC-derived sEVs [91]. This process promotes Ras and PI3K phosphorylation through the inhibition of RASA1, thereby enhancing endothelial cell proliferation and mitigating damage to the BBB [91]. Pericyte-derived sEVs with miR-210 have been shown to enhance BBB function following spinal cord injury by modulating the JAK1/STAT3 signaling pathway, thereby underscoring the potential role of sEVsin neuroprotection [92]. Similarly, prolonged exercise-induced sEVs with miR-532-5p have been demonstrated to restore BBB function in mouse models of Alzheimer’s disease through the downregulation of EPHA4 [93]. Furthermore, the nasal administration of microglial sEVs overexpressing miRNA-124 have been found to improve BBB integrity and reduce neuronal death by modulating neuroinflammation [94]. Additionally, sEVs have the capacity to activate protective signaling pathways, thereby reinforcing BBB integrity. Research indicates that neural stem cell-derived sEVs from induced pluripotent stem cells (hiPSC–NSC–sEVs) can enhance BBB integrity and decrease leukocyte infiltration by activating the PI3K/AKT/MCP-1 signaling pathway in astrocytes thus improving neurological function following cerebral hemorrhage [95]. Moreover, sEVs possess the ability to influence the expression levels of genes related to the stability of the BBB. In a rodent model of traumatic brain injury (TBI), the administration of sEVs significantly upregulated the expression of the genes associated with BBB stability, featuring occludin, claudin-5, TJP1, RUNX1, and LAMB [91].

### 3.3. Neuroinflammatory

Within the vascular dementia model, it is commonly observed that the stimulation of microglia and astrocytes is dysregulated, leading to an upregulation of pro-inflammatory factors and impaired neural tissue repair [96,97,98]. sEVs are essential in modulating neuroinflammatory processes, thereby aiding in the alleviation of vascular dementia (Figure 2) (Table 3). Research indicates that sEVs have the potential to mitigate the neuroinflammation induced by M1 microglia. For instance, bone marrow mesenchymal stem cell-derived sEVs (BMSCs-sEVs) enriched with miR-146a-5p have been shown to inhibit M1 microglial polarization by downregulating the expression of NFAT5 and IRAK1 [99]. Human umbilical cord mesenchymal stem cell-derived sEVs, specifically containing miR-146a-5p, mitigate neuroinflammation mediated by microglia through the suppression of the IRAK1/TRAF6 signaling pathway [100]. Additionally, mesenchymal stem cell-derived sEVs overexpressing microRNA-223-3p alleviate cerebral ischemia/reperfusion injury by inhibiting the CysLT2R-mediated signaling pathway and reducing the pro-inflammatory response associated with microglial M1 polarization [101]. Moreover, sEVs have the capacity to target critical inflammatory signaling pathways to mitigate neuroinflammation. Notably, the NF-κB pathway is a pivotal signaling mechanism involved in the regulation of various pro-inflammatory cytokines [102,103,104]. Studies have demonstrated that, in the context of treating certain neurodegenerative diseases, sEVs can attenuate the suppression of pro-inflammatory factor release by inhibiting the NF-κB pathway, thereby diminishing the neuroinflammatory response [105,106,107,108]. For example, umbilical cord mesenchymal stem cell-derived sEVs have been shown to inhibit the NF-κB/MAPK signaling pathway and attenuate the inflammatory response thus facilitating the recovery process in spinal cord injury [105]. In an Alzheimer’s disease (AD) mouse model, bone marrow-derived mesenchymal stem cell (BM-MSC)-derived sEVs downregulate NF-κB in the astrocytes through miR-146a, thereby reducing inflammation, enhancing synaptogenesis, and ameliorating the cognitive deficits in the AD mice [106]. Bone marrow mesenchymal stromal cell-derived sEVs mitigate neuroinflammation through the inhibition of the TLR2/IRAK1/NF-κB signaling pathway, consequently enhancing the M2/M1 microglial ratio [107]. Similarly, hypoxia-preconditioned mesenchymal stem cell-derived sEVs with miR-216a-5p exert anti-inflammatory effects by downregulating the TLR4/NF-κB/PI3K/AKT signaling pathway, thereby facilitating the polarization of microglia from the M1 to the M2 phenotype [108]. Moreover, adipose stem cell-derived sEVs with miR-188-3p have the potential to confer neuroprotection by inhibiting the autophagy mediated by cyclin-dependent kinase 5 (CDK5) and the inflammation mediated by NLRP3, as demonstrated in recent studies [109].

### 3.4. Oxidative Stress

Oxidative stress is a critical factor in the pathogenesis of vascular dementia. It arises from an imbalance characterized by the excessive production or diminished scavenging capacity of free radicals [110]. This imbalance is widely recognized as a fundamental pathological mechanism underlying various neurodegenerative diseases, including Alzheimer’s disease (AD) and vascular dementia [111,112]. sEVs are crucial in modulating oxidative stress, thereby contributing positively to the amelioration of vascular dementia (Figure 2) (Table 4). Initially, sEVs mitigate oxidative stress through the regulation of specific genes and signaling pathways. For instance, research indicates that mesenchymal stem cell-derived sEVs can markedly diminish oxidative stress and neuroinflammation by modulating the NRF2/NF-κB/NLRP3 signaling pathway, consequently enhancing neurological function [113]. Human umbilical cord mesenchymal stem cell-derived sEVs (hucMSC-sEVs) have been shown to mitigate oxidative stress and apoptosis through the GPX1-mediated neutralization of hydrogen peroxide [114]. Furthermore, serum-derived sEVs containing miR-137 have the potential to influence neuronal oxidative stress in Parkinson’s disease by modifying OXR1 expression [115]. Moreover, the miRNA constituents within sEVs may play a crucial role in the regulation of oxidation-induced stress. As an illustration, mesenchymal stem cell-derived sEVs can transport specific miRNAs, such as miR-125a-5p, which are capable of inhibiting the endoplasmic reticulum stress induced by high glucose levels [116]. Hippocampal neural stem cell-derived sEVs have been shown to ameliorate the cognitive deficits in rat models of vascular dementia mediated by the miR-34b-5p/CALB1 signaling pathway, which is fundamental to modulate oxidative stress [117]. Similarly, cardiac progenitor cell-derived sEVs with a high concentration of miR-935 have demonstrated a protective effect on cardiomyocytes, safeguarding them against the apoptosis and necrosis induced by oxidative stress, thus mitigating oxidative stress-related damage to some extent [118]. Furthermore, sEVs possess the capability to mitigate oxidative stress by transporting antioxidant enzymes and other antioxidant molecules. As an example, induced pluripotent stem cell-derived sEVs have been proven to convey Necrostatin-1, which alleviates oxidative stress and mitochondrial dysfunction in heart failure by modulating the PARP1/AIFM1 axis [119]. Additionally, certain sEVs are capable of delivering antioxidants such as glutathione, thereby contributing to the reduction in reactive oxygen species levels within cells and diminishing oxidative stress-induced neuronal damage [120]. sEVs also possess the ability to mediate antioxidant effects by transporting mitochondrial NAD-dependent deacetylase sirtuin-3 (SIRT3) [121].

## 4. The Potential of sEVs in the Diagnosis and Treatment of Vascular Dementia

### 4.1. sEVs as Diagnostic Markers

The exploration of sEVs as non-invasive biomarkers for vascular dementia represents a field of ongoing research. sEVs isolated from blood or cerebrospinal fluid (CSF) have the potential to mirror the pathological conditions of the brain and vasculature [122,123]. Alterations in the concentrations of specific sEVs components, including particular proteins or microRNAs, have been correlated with the advancement of vascular dementia. For instance, Yang et al. identified an elevation in serum sEV-associated miR-135a levels and a reduction in miR-193b levels in patients diagnosed with vascular dementia [124]. Zhao et al. observed an elevation in plasma sEV-miRNA-223-3p levels among patients diagnosed with cerebral small vessel disease, noting a significant upregulation of miRNA-223-3p expression concomitant with the onset of cognitive impairment [125]. Additionally, another study reported a marked reduction in the serum sEV-associated miR-23a, miR-29a, and miR-130b levels among the subjects with vascular dementia compared to the healthy controls [126]. In patients with vascular dementia, the circulating sEV-associated miRNA-154-5p was found to be upregulated, a finding that was corroborated in a rat model of vascular dementia caused by vascular occlusion. This upregulation of miR-154-5p was observed to significantly impair endothelial progenitor cell (EPC) function and inhibit angiogenesis in the vascular dementia rat model [12]. Furthermore, Elahi and his colleagues discovered that, in comparison to individuals without white matter hyperintensity, the concentrations of certain proteins, including LAT1 and GLUT1, were significantly elevated in the plasma sEVs of patients suffering from cSVD or endothelial disease [127]. Subsequent research has demonstrated that sEVs are detectable in various body fluids, including blood, urine, and cerebrospinal fluid, thereby offering the potential for their use as non-invasive diagnostic tools [128]. In the context of Alzheimer’s disease research, sEVs have been employed to identify early molecular alterations, a technique that could similarly be applied to the early diagnosis of vascular dementia [129]. Overall, sEVs exhibit significant promise as diagnostic markers for vascular dementia. Future investigations should aim to identify specific sEV markers across different dementia types to facilitate more precise diagnoses and personalized therapeutic approaches.

### 4.2. sEVs as Vehicles for Drug Delivery

sEVs represent a promising medication delivery method for the administration of therapeutic agents and pharmaceuticals [130]. Their intrinsic nano-scale dimensions and membrane-encapsulated architecture confer protection against drug degradation and metabolism, thereby enhancing the stability and targeting efficiency of drugs in vivo [131,132,133]. Furthermore, the sEV surface can be modified and functionalized to facilitate the targeted delivery and controlled release of drugs, thereby augmenting therapeutic efficacy [134,135,136]. sEVs present numerous advantages as drug delivery vehicles. Firstly, they possess the capability to efficiently encapsulate and safeguard pharmaceutical agents from degradation or metabolic processes within the body, thereby enhancing the stability and bioavailability of these drugs [137,138]. Secondly, the nano-scale dimensions and membrane-encapsulated architecture of sEVs support the delivery of drugs to designated cells or tissues via mechanisms such as cell membrane fusion or receptor-mediated endocytosis [139,140]. This targeted approach has the potential to elevate the local concentration of the drug while minimizing the adverse effects on non-target tissues [139,141,142]. Furthermore, sEVs have the capability to modulate the physiological functions and signaling pathways of cells through interactions with the target cells, thereby enhancing the therapeutic efficacy [143,144]. Applying sEVs in the management of neurological diseases has demonstrated significant potential. Research indicates that sEVs are capable of traversing the blood–brain barrier to deliver therapeutic agents to the central nervous system, thereby ameliorating the symptoms associated with neurodegenerative conditions such as Alzheimer’s disease [145,146]. Furthermore, sEVs serve as effective carriers for anti-inflammatory drugs in the management of neuroinflammation-related disorders [147]. In the context of vascular dementia, sEVs can mitigate the neuronal damage induced by cerebrovascular disease through the delivery of antioxidants or anti-inflammatory molecules. Studies have revealed that sEVs confer neuroprotective effects by modulating neuronal survival and function [148,149]. Additionally, sEVs can influence the pathological processes of vascular dementia by transporting specific microRNAs or proteins [12]. In summary, sEVs hold considerable promise as drug delivery vehicles in the treatment of vascular dementia. Future investigations should aim to elucidate the precise mechanisms and application strategies of sEVs across various nervous system disorders to facilitate the advancement of more efficacious remedial interventions.

## 5. Challenges and Limitations of sEV-Based Therapies

### 5.1. Standardization and Normalization Issues

As a novel therapeutic modality, sEVs confront challenges in the standardization and regularization of their clinical applications. In the intricate processes of sEV isolation, purification, and quality control, the lack of a standardized framework constitutes a remarkable impediment [150]. sEVs are commonly isolated from diverse biological samples, such as cell culture supernatants, blood, and other bodily fluids [151]. Currently, a wide variety of isolation techniques are available, including ultracentrifugation, size-exclusion chromatography, and immunoaffinity-based methods [152]. For example, ultracentrifugation, a widely used technique, is both time-consuming and may cause damage to sEVs due to the high-speed centrifugal forces [153,154]. Size-exclusion chromatography can yield relatively pure sEVs but demands sophisticated equipment and careful calibration [155,156]. Apart from isolation, the purification of sEVs also lacks standardization. After isolation, subsequent purification steps are essential for eliminating residual contaminants. Some laboratories may adopt additional filtration steps, like using a 0.22 μm filter to remove larger particles [157], while others may rely on precipitation methods, such as using polyethylene glycol to precipitate sEVs [158]. These different treatment approaches result in variances in the purity levels of sEV preparations, thereby influencing the interpretation of experimental results. The quality control measures for sEVs remain in a state of disorder. Well-established, standardized criteria for assessing the quality of sEV preparations have not been established yet. Parameters such as sEV concentration (which can be measured by nanoparticle tracking analysis), size distribution, and the presence or absence of contaminants should be precisely defined and measured [159,160,161]. Without standardized quality control, it is extremely arduous to compare the results of different studies. In summary, the establishment of unified standards for sEV isolation, purification, and quality control is of crucial importance for promoting the clinical translation of sEV-based therapies. Although the International Society for Extracellular Vesicles (ISEV) has made efforts in formulating some general guidelines, much more work is required to fully standardize these aspects of sEV research [162].

### 5.2. Production and Storage Challenges

The development of large-scale and highly efficient production and storage technologies for small extracellular vesicles represents a significant challenge in their therapeutic applications. Currently, the preparation methods of small extracellular vesicles (sEVs) are faced with the limitations of high cost and low yield [163]. Ultracentrifugation is a widely used technique. Although it can obtain sEVs, this method is time-consuming, requires expensive equipment, and has a relatively low yield [164]. To address the bottlenecks in large-scale production, researchers are exploring a variety of innovative strategies. One approach is the use of microfluidic devices. These devices have the potential to achieve the continuous and high-throughput production of sEVs and can better control the separation process [165]. By precisely controlling the liquid flow in the microchannels, sEVs can be effectively separated from complex biological mixtures [166]. Moreover, compared with traditional methods, microfluidic systems require less starting material, making them more cost-effective in large-scale production [35]. Another approach is to engineer the extracellular environment to enhance the secretion and function of small extracellular vesicles [167]. For instance, research has demonstrated that magnetic iron oxide nanoparticles can enhance the production of sEVs by upregulating the genes associated with the transportation and secretion of sEVs [168]. Meanwhile, the application of 3D cell culture systems is also being explored. These systems can better mimic the in vivo microenvironment, promoting cell growth and sEV production [169]. They have the potential to improve the yield of sEVs while maintaining their quality and function [170]. Furthermore, the issues regarding the storage and stability of sEVs also need to be addressed. Currently, the storage method at −80 °C is the most commonly adopted approach [171]. Although this extremely low temperature helps to slow down biochemical reactions and maintain the integrity of sEVs to a certain extent, it requires the use of specialized and energy-consuming equipment, such as ultra-low-temperature freezers [172]. Additionally, even under the storage condition of −80 °C, long-term storage may still affect the integrity and function of sEVs [173]. Over time, some studies have shown that the bioactive molecules associated with sEVs may gradually lose their activity thus leading to the loss of potential therapeutic effects [172]. To solve these storage-related problems, some researchers are exploring the use of cryoprotectants. These substances can be added to the sEV suspension to protect sEVs from damage during the freezing and thawing processes [174]. Another emerging method is lyophilization, which involves removing water through freeze-drying and converting sEVs into a dry and stable form [175]. This technology has the potential to extend the shelf-life of sEVs and simplify their storage and transportation. However, the use of cryoprotectants and lyophilization technology are still in the experimental stage, and a large amount of research is still needed to optimize these methods.

### 5.3. Regulatory and Ethical Concerns

The application of sEVs, especially those derived from stem cells or engineered for gene delivery, has given rise to a multitude of regulatory and ethical considerations that demand careful attention. On the one hand, when using sEVs derived from stem cells, there may be risks of tumorigenesis and immune rejection. Although sEVs themselves have relatively low immunogenicity, the bioactive molecules they carry may trigger an immune response [176]. In allogeneic transplantation, the recipient’s immune system may recognize the sEVs from donor stem cells as foreign substances, leading to a rejection reaction [177,178]. Additionally, if the immunomodulatory effect of sEVs is excessive, it may result in an over-suppression of the immune system, increasing the risks of infection and tumorigenesis [179]. On the other hand, engineered sEVs for gene delivery must strictly adhere to regulatory requirements to ensure their safety and efficacy. These sEVs are designed to carry genetic materials such as DNA or RNA and deliver them to specific cells to modify gene expression [180]. However, this process must undergo rigorous pre-clinical and clinical trials to verify its safety and effectiveness [181]. Meanwhile, ethical issues such as the source of cells for sEV production and potential off-target effects also need to be resolved. When using human cells as the source, strict ethical standards should be followed to ensure the informed consent of donors and the protection of their privacy [182]. The natural characteristics of sEVs may cause them to accumulate in non-target tissues or organs [183]. Researchers need to ensure that sEV-based therapies can precisely target the intended cells and minimize adverse reactions [184]. Currently, regulatory authorities are still in the process of formulating comprehensive guidelines for sEV-based therapies. More research and discussions are required to establish clear ethical and regulatory frameworks.

### 5.4. Delivery-Related Challenges

sEVs can be delivered through various routes, each with its own advantages and limitations. Intravenous (IV) delivery is a common and convenient method. Given that sEVs can cross the blood–brain barrier (BBB), IV delivery has the potential to reach the brain and treat vascular dementia [185]. However, it is not entirely clear if IV delivery is sufficient. After an intravenous injection, sEVs may be rapidly cleared from the circulation by the reticuloendothelial system (RES), reducing their availability to reach the target site in the brain [186,187]. To enhance the effectiveness of IV-delivered sEVs, strategies such as surface modification can be employed. For example, coating sEVs with polyethylene glycol (PEG) can increase their circulation time by preventing RES uptake [188]. Additionally, conjugating targeting ligands to the sEV surface can improve their specificity for brain-related cells, enhancing their ability to cross the BBB and reach the affected areas in the brain [189]. Another potential route is intranasal delivery. This route allows sEVs to bypass the BBB to some extent and directly reach the central nervous system through the olfactory nerve pathway [190]. Intranasal delivery has shown promise in pre-clinical studies, as it can deliver sEVs to the brain with relatively high efficiency and may reduce the systemic side effects associated with IV delivery [191,192,193]. However, challenges remain in optimizing the formulation for intranasal delivery to ensure proper absorption and distribution within the nasal cavity and subsequent transport to the brain. Local delivery methods, such as direct injection into the brain parenchyma or into the cerebrospinal fluid (CSF), can also be considered [194,195,196]. These methods can achieve high local concentrations of sEVs at the target site, but they are invasive and carry risks such as infection, tissue damage, and potential immune responses at the injection site. Therefore, the careful consideration of the benefits and risks is required when choosing a delivery route for sEV-based therapies in vascular dementia treatment.

## 6. Conclusions and Future Directions

sEVs present substantial potential as diagnostic biomarkers and therapeutic agents for vascular dementia. Their capacity to mirror disease status and deliver targeted therapy positions them as valuable instruments in the management of vascular dementia. However, numerous challenges, including issues related to standardization, production, storage, regulation, and delivery, need to be overcome. Future research should focus on addressing these challenges. This includes developing standardized methods for sEV isolation, purification, and quality control; exploring new production technologies to increase yields and reduce costs; studying optimal storage conditions to maintain sEV stability; establishing clear regulatory and ethical guidelines for sEV-based therapies; and further investigating effective delivery routes. Further investigation of the role and mechanisms of sEVs in vascular dementia will provide a theoretical and experimental basis for the development of novel diagnostic methods and therapeutic strategies. Ongoing research in sEV biology, alongside advancements in scientific technology, may yield more effective solutions to this complex issue. sEV therapy represents an innovative therapeutic approach aimed at preventing or decelerating the progression of vascular dementia. This strategy holds the potential to provide more efficacious treatment options for individuals afflicted with vascular dementia, thereby enhancing their quality of life.

## Figures and Tables

**Table 1:** Mechanisms and Research Advances of sEVs in Alleviating Vascular Injury for the Treatment of Vascular Dementia.

Source of sEVs	Key Components	Signaling Pathways/Mechanisms	Target Cells/Targets	Functions/Effects	References
Mesenchymal Stem Cells (MSCs)	Growth factors (EGF, FGF, PDGF)	Enhancing endothelial cell proliferation and migration	Endothelial cells	Promoting angiogenesis	[77]
Human Umbilical Cord Blood	miR-21-3p	Activating PI3K/Akt and ERK1/2 pathways	Endothelial cells	Promoting wound healing and stimulating neovascularization	[78]
SHED Aggregates (SA-sEVs)	miR-26a	Regulating TGF-β/SMAD2/3 signaling pathway	SHED cells and HUVECs	Promoting angiogenesis and enhancing endothelial cell functions	[79]
MSC-derived sEVs	ICAM1	Activating SMAD3 and P38MAPK pathways	HBMECs	Enhancing angiogenesis	[80]
CD133+ Cells	mRNAs (e.g., kit ligand, IGF-1, VEGF, FGF, IL-8)	Directly provide pro-angiogenic factors	HUVECs	Promoting angiogenesis both in vitro and in vivo	[81]
MSC-derived sEVs	miRNAs (e.g., miR-30b, miR-30c, miR-424, let-7f)	Upregulate the expression of pro-angiogenic factors	Endothelial cells	Enhancing pro-angiogenic factor expression and stimulating neovascularization	[82]
Endothelial Cells	miRNA (miR-214)	Inhibit cellular senescence and promote angiogenesis	Endothelial cells	Delaying cellular aging and enhancing angiogenesis	[83]

**Table 2:** Mechanisms and Research Advances of sEVs in Alleviating BBB Dysfunction for the Treatment of Vascular Dementia.

Source of sEVs	Key Components	Signaling Pathways/Mechanisms	Target Cells/Targets	Functions/Effects	References
Mesenchymal Stem Cells (MSCs)	miR-132-3p	Inhibition of RASA1, activation of Ras and PI3K phosphorylation	Endothelial cells	Promotes endothelial cell proliferation, restores BBB structure, and mitigates BBB damage	[91]
Pericytes	miR-210	Regulation of the JAK1/STAT3 signaling pathway	BBB	Enhances BBB function and stabilizes the BBB after spinal cord injury	[92]
Exercise-induced sEVs	miR-532-5p	Downregulation of EPHA4 expression	BBB	Restores BBB function in Alzheimer’s disease mouse models	[93]
Microglia	miR-124	Regulation of neuroinflammation	BBB, Neurons	Improves BBB integrity and reduces neuronal death	[94]
Neural Stem Cells Derived from Induced Pluripotent Stem Cells (hiPSC-NSCs)	sEVs	Activation of the PI3K/AKT/MCP-1 signaling pathway	Astrocytes	Enhances BBB integrity, reduces leukocyte infiltration, and improves neurological function after cerebral hemorrhage	[95]
Mesenchymal Stem Cells (MSCs)	Genes such as occludin, claudin-5, TJP1, RUNX1, and LAMB	Upregulation of genes related to BBB stability	BBB	Improves BBB stability and mitigates vascular damage caused by traumatic brain injury	[91]

**Table 3:** Mechanisms and Research Advances of sEVs in Alleviating Neuroinflammation for the Treatment of Vascular Dementia.

Source of sEVs	Key Components	Signaling Pathways/Mechanisms	Target Cells/Targets	Functions/Effects	References
Bone Marrow Mesenchymal Stem Cells (BMSCs)	miR-146a-5p	Downregulation of NFAT5 and IRAK1 expression, inhibition of M1 microglial polarization	M1 microglia	Suppresses neuroinflammatory responses	[99]
Umbilical Cord Mesenchymal Stem Cells (UC-MSCs)	miR-146a-5p	Inhibition of IRAK1/TRAF6 signaling pathway	Microglia	Mitigates microglia-mediated neuroinflammation	[100]
Mesenchymal Stem Cells (MSCs)	miR-223-3p	Inhibition of CysLT2R signaling pathway, reduction in pro-inflammatory response related to M1 microglial polarization	M1 microglia	Alleviates cerebral ischemia/reperfusion injury	[101]
Umbilical Cord Mesenchymal Stem Cells (UC-MSCs)	sEVs	Inhibition of NF-κB/MAPK signaling pathway	BV2 microglia and rat spinal cord tissues	Reduces inflammatory response and promotes recovery after spinal cord injury	[105]
Bone Marrow Mesenchymal Stem Cells (BM-MSCs)	miR-146a	Inhibition of NF-κB signaling pathway	Astrocytes	Reduces astrocytic inflammation, enhances synaptogenesis, and improves cognitive deficits in Alzheimer’s disease	[106]
Bone Marrow Mesenchymal Stem Cells (BM-MSCs)	sEVs	Inhibition of TLR2/IRAK1/NF-κB signaling pathway, increases M2/M1 microglial ratio	Microglia	Suppresses neuroinflammatory responses	[107]
Hypoxia-Preconditioned Mesenchymal Stem Cells (H-MSCs)	miR-216a-5p	Inhibition of TLR4/NF-κB/PI3K/AKT signaling pathway, promotes M1-to-M2 microglial polarization	Microglia	Suppresses neuroinflammation and enhances anti-inflammatory effects	[108]
Adipose-Derived Stem Cells (ASCs)	miR-188-3p	Inhibition of autophagy mediated by CDK5 and inflammation mediated by NLRP3	PD mice and MN9D cells	Provides neuroprotection and reduces inflammation	[109]

**Table 4:** Mechanisms and Research Advances of sEVs in Reducing Oxidative Stress for the Treatment of Vascular Dementia.

Source of sEVs	Key Components	Signaling Pathways/Mechanisms	Target Cells/Targets	Functions/Effects	References
Mesenchymal Stem Cells (MSCs)	NRF2, NF-κB, NLRP3	Modulation of NRF2/NF-κB/NLRP3 pathway	Microglia	Reduces oxidative stress and neuroinflammation; enhances neurological function	[113]
Human Umbilical Cord MSCs (hucMSC-sEVs)	GPX1	Neutralization of hydrogen peroxide	Hepatocytes	Mitigates oxidative stress and apoptosis	[114]
Serum-derived sEVs	miR-137	Regulation of OXR1 expression	Neurons	Modulates neuronal oxidative stress in Parkinson’s disease	[115]
MSC-derived sEVs	miR-125a-5p	Inhibition of endoplasmic reticulum stress induced by high glucose	Corneal epithelial cells	Prevents oxidative damage to corneal epithelial cells	[116]
Hippocampal Neural Stem Cells	miR-34b-5p	miR-34b-5p/CALB1 signaling pathway	Neurons	Ameliorates cognitive deficits by reducing oxidative stress	[117]
Cardiac Progenitor Cells	miR-935	Protection against apoptosis and necrosis	Cardiomyocytes	Mitigates oxidative stress-related damage to cardiomyocytes	[118]
Induced Pluripotent Stem Cells (iPSCs)	Necrostatin-1	Modulation of PARP1/AIFM1 axis	Cardiomyocytes	Reduces oxidative stress and mitochondrial dysfunction	[119]
sEVs containing antioxidants	Glutathione	Reduction in reactive oxygen species (ROS) levels	Neurons	Decreases oxidative stress-induced neuronal damage	[120]
sEVs transporting SIRT3	SIRT3	Antioxidant effects mediated by SIRT3	SH-SY5Y cells	Protects cells by reducing oxidative stress	[121]

## Data Availability

No new data were created or analyzed in this study.
